# Effects of GLP-1 and Incretin-Based Therapies on Gastrointestinal Motor Function

**DOI:** 10.1155/2011/279530

**Published:** 2011-06-22

**Authors:** Chinmay S. Marathe, Christopher K. Rayner, Karen L. Jones, Michael Horowitz

**Affiliations:** ^1^Discipline of Medicine, Royal Adelaide Hospital, University of Adelaide, Adelaide SA 5000, Australia; ^2^Centre of Clinical Research Excellence in Nutritional Physiology, Interventions and Outcomes, University of Adelaide, Adelaide SA 5000, Australia

## Abstract

Glucagon-like peptide 1 (GLP-1) is a hormone secreted predominantly by the distal small intestine and colon and released in response to enteral nutrient exposure. GLP-1-based therapies are now used widely in the management of type 2 diabetes and have the potential to be effective antiobesity agents. Although widely known as an incretin hormone, there is a growing body of evidence that GLP-1 also acts as an enterogastrone, with profound effects on the gastrointestinal motor system. Moreover, the effects of GLP-1 on gastrointestinal motility appear to be pivotal to its effect of reducing postprandial glycaemic excursions and may, potentially, represent the dominant mechanism. This review summarizes current knowledge of the enterogastrone properties of GLP-1, focusing on its effects on gut motility at physiological and pharmacological concentrations, and the motor actions of incretin-based therapies. While of potential importance, the inhibitory action of GLP-1 on gastric acid secretion is beyond the scope of this paper.

## 1. Introduction

A role for gastrointestinal peptides (or factors) in the maintenance of mammalian glucose homeostasis had been speculated for more than 100 years. The search for these gut peptides was stimulated initially by the discovery of secretin by Bayliss and Starling in 1902 [1] and advanced by the work of others [2–5] before it fell out of favour. It was revived subsequently by the observations, by Elrick et al. [6] and McIntyre et al. [7] in 1964, that an oral glucose load resulted in a much greater insulin response than an intravenous glucose load despite resulting in comparable blood glucose concentrations—the so-called “incretin effect” [8]. Glucagon-like peptide-1 (GLP-1) was discovered in the 1980s following sequencing of the proglucagon gene and was shown soon after that time to have fulfilled the physiological criteria for an “incretin” as specified by Creutzfeldt [9], that is, a hormone released from intestinal cells following a nutrient load, which leads to a glucose dependent insulin response. GLP-1 was the second incretin to be characterized after glucose-dependent insulinotropic polypeptide (GIP), which had been discovered a decade earlier.

**Figure 1:**
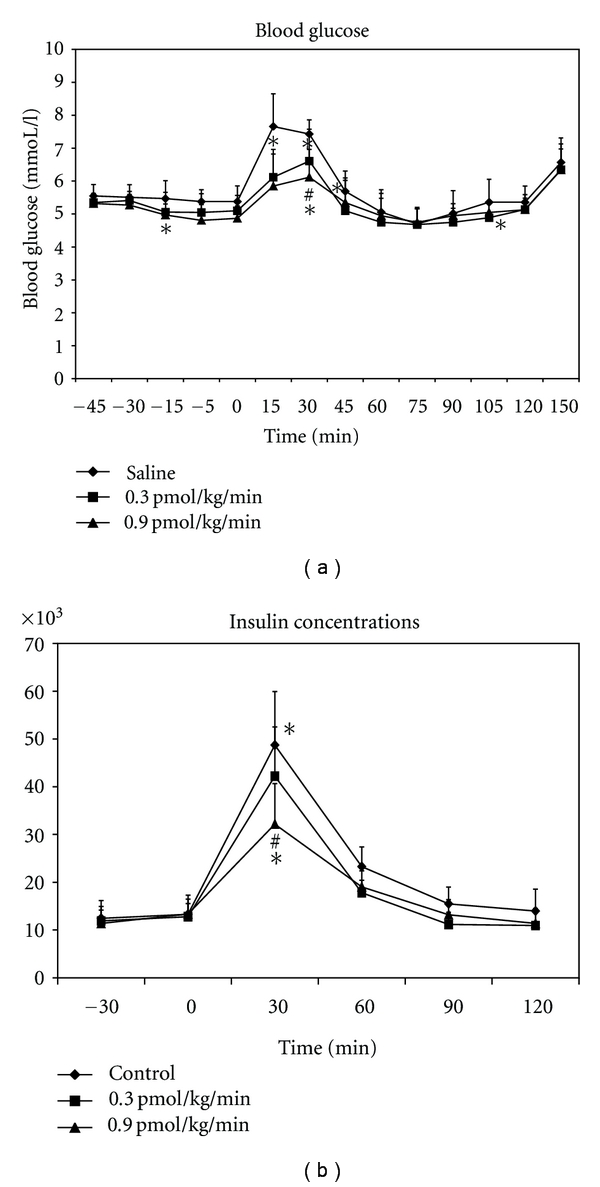
Blood glucose (a) and plasma insulin (b) concentrations during IV infusion of GLP-1, at 0.3 and 0.9 pmol/kg/minutes, or 0.9% saline, in 10 healthy humans. Data are means ± SEM. *GLP-1 0.3 and 0.9 versus saline: *P* < .05; ^#^GLP-1 0.9 versus GLP-1 0.3: *P* < .05 (adapted from Little et al. 2006 [19], and used with permission from the publisher).

GLP-1, however, does not fit as well as GIP within Creutzfeldt's incretin definition [10]. For example, in healthy subjects [11] and type 2 diabetic patients [12], postprandial (as opposed to fasting) levels of insulin and C peptide are *decreased* by exogenous GLP-1, rather than stimulated (Figure 1), and when the slowing of gastric emptying induced by GLP-1 is reversed by the prokinetic drug erythromycin, the glucose lowering effect is attenuated [13]. The properties of GLP-1 as an enterogastrone (i.e., a factor that slows gastric emptying and inhibits gastric acid secretion) have also been appreciated [10, 14, 15]. In fact, it has been suggested that the actions of GLP-1 to slow gastric emptying, and thereby the entry of nutrients into the small intestine to delay their absorption, may outweigh its insulinotropic and glucagonostatic effects [16]. In contrast to GLP-1, GIP has little effect on gastric emptying [17]. If anything, there is some evidence that GIP may modestly accelerate emptying from the stomach [18]. 

Some studies have reported reduced GLP-1 levels in type 2 diabetic patients [18, 20–24], although this has not been found uniformly to be the case [25, 26]. It should be noted, however, that the efficacy of GLP-1-based therapy does not depend on a deficiency of endogenous peptide. Pharmacological “replacement” of GLP-1 is not straightforward owing to its very short half-life. GLP-1 is rapidly degraded by the enzyme dipeptidyl peptidase-4 (DPP-4), making it impractical for most clinical applications. This has provided the rationale for the development, and subsequent widespread use, of synthetic longer-acting analogues, such as exenatide and liraglutide (the two approved GLP-1 analogues), and DPP-4 inhibitors, like sitagliptin and vildagliptin, to improve glycaemic control in type 2 diabetic patients [27].

## 2. The Physiology of Gastrointestinal Motility

An overview of the physiology of gastrointestinal motility is useful in understanding the role and importance of GLP-1 in gut function. Gastric emptying, a highly regulated process of delivering chyme from the stomach to the small intestine, involves a complex interplay between the gastrointestinal smooth muscle, gastric pacemaker cell networks, the so-called interstitial cells of Cajal, and neurohormonal systems, particularly inhibitory feedback arising from the interaction of nutrients with the small intestine. Gastric and small intestinal motility is of predominutesantly two types: (a) peristaltic, in the interdigestive or fasted phase, and (b) segmented contractions in the fed, or postprandial state. Interdigestive motility is comprised of three sequential phases (phase I or quiescent, lasting *∼*40 minutesutes, phase II or intermittent, lasting *∼*50 minutesutes, and phase III or regular contractions, at about 3/minutesute in the stomach and 10–12/minutesute in the small intestine, and lasting *∼*5–10 minutesutes) and is called the “migrating motor complex” (MMC) [28]. Transit of indigestible solid occurs mainly in late phase II and phase III of the MMC [29]. 

Once food (solid, liquid, or mixed) arrives in the stomach, the MMC is replaced by the postprandial motor pattern. For a solid or mixed meal, the proximal and distal parts of the stomach have different functions. The proximal stomach is concerned with the storage of food and accommodates the ingested meal by decreasing its tone [30, 31], enabling its volume to increase without a substantial increase in intragastric pressure [31, 32]. In contrast, the distal portion of the stomach is concerned with the mixing and trituration of the meal. Antral contractions pulverise the digestible solid contents against the closed pylorus until they have achieved a size of 1-2 mm [33]. Phasic and tonic contractions localised to the pylorus play a major role in the regulation of gastric emptying so food particles are delivered to the duodenum following the opening of the pylorus, predominutesantly in a pulsatile manner [34]. The rate and pattern of gastric emptying are dependent on the composition (solid, semisolid, or liquid), osmolarity, caloric content, and size of the particles in the meal ingested. Liquids are preferentially transferred to the small intestine before solids. Nonnutrient liquids empty in an overall exponential pattern, while a more linear rate is observed as the nutrient and caloric content of the liquid meal increase. The presence of food in the stomach reduces appetite, and distension of the antrum, rather than the proximal stomach, appears to be more strongly associated with the perception of postprandial fullness [35], and suppression of subsequent energy intake [36]. 

The exposure of the small intestine to nutrients triggers a powerful inhibitory feedback to slow gastric emptying and small intestinal transit—the magnitude of this feedback is dependent on the type of nutrient [37], as well as both the length [38–40] and region [40–42] of small intestine exposed. GLP-1 and peptide YY (PYY), coexpressed with GLP-1 in the entero-endocrine L cells predominutesantly (but not exclusively) from the distal gut, appear to be potent mediators of the so-called “ileal brake” [43]. This small intestinal feedback mechanism results in highly regulated gastric emptying of nutrients, including carbohydrate, from the stomach to the small intestine at an overall rate of about 1 to 4 kcal/minutes [44, 45]. The release of GLP-1 from the small intestine is critically dependent on the carbohydrate load in both healthy subjects and type 2 diabetic patients [45–48]. When glucose is infused intraduodenally at the rate of 1 kcal/minutes there is a minutesimal, and transient, release of GLP-1, whereas there is a substantial, and sustained, GLP-1 response to infusion at the rate of 4 kcal/minutes [49], probably reflecting a greater length of small intestinal exposure [38]. Fat (mediated by free fatty acids), protein [50], and bile acids [51] are also potent stimuli of GLP-1 release from the L cells. For carbohydrate, it has been suggested that stimulation of intestinal “sweet taste” receptors triggers GLP-1 release [52], although in humans, the artificial sweetener, sucralose, does not induce GLP-1 secretion [53]. However, secretion of GLP-1 in response to sucrose is increased when malabsorption is induced by the *α*-glucosidase inhibitor, acarbose, presumably due to exposure of the L cell—bearing distal gut to larger amounts of carbohydrate [54]. 

The terminutesal aspect of the gastrointestinal tract, the colon, is characterized by the presence of haustra (formed by nonpropagated phasic contractions), which compartmentalise the luminutesal compartment and favour stool formation by water and electrolyte absorption. Colonic motility is discontinuous, and mostly slow, which ensures prolonged retention of contents, though occasionally rapid activity allows large amounts of residue to be transferred across the colon within seconds. Expulsion of contents is regulated by the ano-rectum.

## 3. The Interrelationship of Gastric Emptying with Postprandial Glycaemia

**Figure 2:**
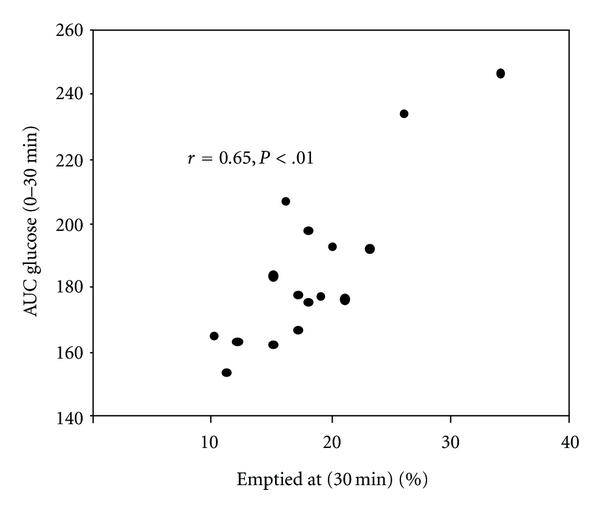
Relationship between the area under the plasma glucose concentration curve between 0 and 30 minutes and the retention of the meal in the stomach at 30 minutes (*r* = 0.65, *P* < .01) (adapted from Horowitz et al. 1993 [45] and used with the permission of the publisher).

Postprandial hyperglycaemia is being increasingly recognised as an independent predictor of cardiovascular mortality in both diabetic and nondiabetic populations [55, 56]. The rate of gastric emptying influences postprandial glycaemic excursions and vice versa: a situation comparable to the “chicken and egg” relationship [57]. Gastric emptying is a major determinutesant of postprandial glycaemic excursions in healthy subjects [45] as well as type 1 and type 2 diabetic patients [46] so that slower gastric emptying is associated with reduction in blood glucose, especially in the first hour after ingestion of a meal [58, 59] (Figure 2). Conversely, gastrointestinal motor function is highly sensitive to changes in the glycaemic state [57]. For example, in both type 1 [60] and type 2 diabetic patients [61], an increase in postprandial blood glucose levels is associated with proportional slowing of gastric emptying. Even changes within the physiological postprandial blood glucose range (i.e., 4 mmol/L versus 8 mmol/L) affect gastric emptying in healthy as well as uncomplicated type 1 diabetic patients [62]. The slowing of gastric emptying by acute hyperglycaemia reflects the stimulation of pyloric motility [62, 63], suppression of antral motility [64], and reduction in proximal gastric tone [65]. Acute hyperglycaemia also attenuates the ability of erythromycin [66], and probably other prokinetic drugs, to accelerate gastric emptying. In contrast to the effect of hyperglycaemia, insulin-induced hypoglycaemia accelerates gastric emptying substantially, probably representing a counter-regulatory mechanism [67]. 

## 4. Effects on Gastric Motility

It has been well established that GLP-1 slows gastric emptying. The following section reviews the effects of GLP-1 and incretin-based therapies on gastric motility. 

### 4.1. Exogenous GLP-1

**Figure 3:**
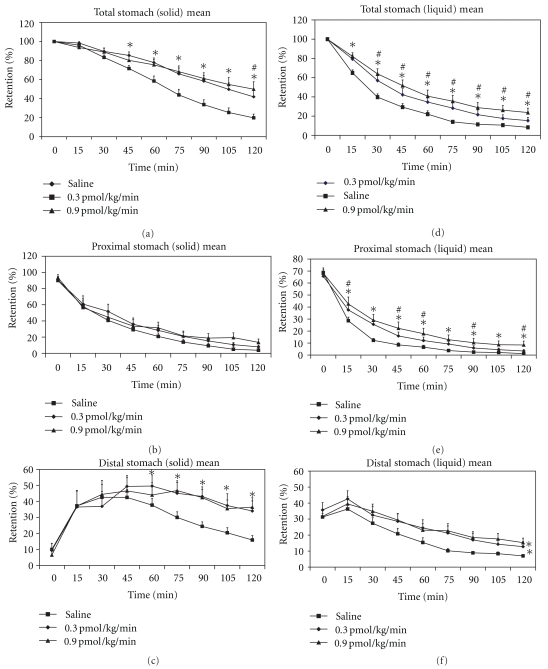
Gastric emptying curves for the solid and liquid components of a meal for the total, proximal, and distal stomach during IV infusion of GLP-1 at 0.3 and 0.9 pmol/kg/minutes, or 0.9% saline, in 10 healthy humans. Data are means ± SEM. *GLP-1 0.3 and 0.9 versus saline: *P* < .05; ^#^GLP-1 0.9 versus GLP-1 0.3: *P* < .05 (adapted from Little et al. 2006 [19] and used with permission from the publisher).

Exogenous GLP-1 slows gastric emptying in healthy [16, 19, 68], obese [69], type 2 diabetic [12], and critically ill subjects [70]. Infusion of GLP-1 slows gastric emptying of both solid and liquid components of a meal and alters intragastric meal distribution so that a greater proportion of the meal is retained in the distal stomach [19] (Figure 3). Even at “low” doses (0.3 pmol/kg/minutes, designed to reflect “physiological” postprandial GLP-1 plasma concentrations) intravenous adminutesistration of GLP-1 profoundly slows gastric emptying in a substantial proportion of healthy subjects into the “gastroparetic” range [19]. In both healthy subjects [16, 19, 71] and type 2 diabetic patients [12], the effect of exogenous GLP-1 on gastric emptying appears to be dose-related. Furthermore, an inverse relationship between the early postprandial rise in blood glucose and the rate of gastric emptying, following infusion of exogenous GLP-1, indicates the importance of the gastric motor actions of GLP-1 in its glucose-lowering effect [19]. As would be predicted by the slowing of gastric emptying, exogenous GLP-1 relaxes the proximal stomach in a dose-dependent manner [72], reduces antral and duodenal motility, and increases pyloric tone in both the fasted and the fed states [71]. 

### 4.2. Endogenous GLP-1

While a number of studies have employed infusions of exogenous GLP-1 at “low” rates, designed to reflect physiological postprandial GLP-1 concentrations, a more valid approach to assess the role of endogenous GLP-1 is the use of a specific GLP-1 antagonist such as exendin (9-39) amide. It appears that endogenous GLP-1 has a modest effect to slow gastric emptying and thereby delay carbohydrate absorption, given a sufficient caloric load [73], by mechanisms that include antral inhibition and stimulation of pyloric motility [34]. Three other studies employing exendin (9-39) failed to show an effect of endogenous GLP-1 on gastric emptying [86–88], but this is likely to reflect methodological differences, including the use of a suboptimal technique (plasma D-xylose) for measuring gastric emptying in one study [87]. Furthermore, in one of these studies [88] although no difference in gastric emptying was observed, exendin 9-39 did change the intragastric distribution of the meal, supporting the role of endogenous GLP-1 in regulating gastric motility. 

### 4.3. GLP-1-Based Therapies

The insulinotropic property of GIP is markedly diminutesished in type 2 diabetic patients [89–91], probably in part as an effect of hyperglycaemia. On the other hand, GLP-1 retains its properties (at supraphysiological doses) in type 2 diabetic patients with potent effects on gastric motility and postprandial glycaemic control [12]. Consequently, this hormone has been an important target for the pharmaceutical industry in the treatment of diabetes [12, 81, 92]. This has stimulated the development of synthetic GLP-1 analogues, which are resistant to rapid degradation, and inhibitors of the enzyme DPP-4 (which boost concentrations of the active fraction of endogenous GLP-1). Both classes of drugs are now used widely in the management of type 2 diabetes. 

#### 4.3.1. GLP-1 Receptor Agonists

There are a number of GLP-1 receptor agonists on the market or in development. Of the established agents, exenatide and liraglutide, the former has been best studied in regards to motor effects. It appears that an important mechanism contributing to the action of exenatide, in reducing postprandial glycaemia, is by slowing gastric emptying [81, 93]. Exenatide, derived from the saliva of the Gila monster *Heloderma suspectum,* has been shown to induce a dose-dependent deceleration of gastric emptying in healthy subjects [80] and type 2 diabetic patients [82, 94]. Exenatide slows gastric emptying of both solid and liquid components of a meal, irrespective of the presence of established autonomic neuropathy [81]. However, in both type 2 diabetic patients [81] and patients with critical illness [70], the effects of GLP-1 or incretin-based therapies appear to be dependent on the prior rate of gastric emptying, so that there is little further slowing in those with delayed emptying at baseline. The relevance of this for selecting the most appropriate patients to be treated with exenatide and other GLP-1 agonists remains to be clarified, but it is clearly an important issue for further study. While animal studies with long acting exenatide (LAR exenatide) have failed to show evidence of tachyphylaxis (i.e., reduction in pharmacological response over time) [95], a recent trial in type 2 diabetic patients indicated that gastric emptying may be more strongly slowed by twice daily exenatide than once weekly LAR exenatide [96], suggesting that continuous GLP-1 exposure could result in a diminutesution of pharmacological response, potentially reflecting changes in receptor activation and/or changes in vagal function.

The major adverse effects of exenatide and liraglutide are nausea and vomiting. While these could relate to its effects on gastric motor function, and antral distension in particular [35, 36, 97], the occurrence of adverse effects seems not to relate closely to the delay in gastric emptying [81, 96], and it is possible that central mechanisms are important. The effects on gastric emptying have not been comprehensively established for incretin-based therapies other than exenatide, but liraglutide also slows gastric emptying—the magnitude of which is uncertain [83, 84]. Evaluation of this aspect of drug action represents an important research priority for these agents.

#### 4.3.2. Dipeptidyl Peptidase-4 Inhibitors

Dipeptidyl peptidase-4 inhibitors, including sitagliptin and vildagliptin, result in an increase in circulating active GLP-1 concentrations [27, 98] but appear to have, at most, a modest effect on gastric emptying [27]. Some deceleration in gastric emptying was observed in a study of obese insulin resistant monkeys treated with vildagliptin [85], but human studies published to date have not demonstrated an effect of DPP-4 inhibitors on the rate of gastric emptying [82, 99, 100], possibly because the elevation in active GLP-1 concentrations is relatively modest. It should be noted that upper gastrointestinal adverse effects such as nausea and vomiting are less commonly encountered with DPP-4 inhibitors than with GLP-1 receptor agonists, and the relative lack of effects of the former on gastric emptying could well be relevant in this regard.

## 5. Effects on Small Intestinal Motility

The effects of GLP-1 on small intestinal motility have not been extensively studied. Exogenous, intravenous GLP-1 has been shown to inhibit murine fasted and fed small bowel motility in a dose-dependent manner and appears to have an additive effect when combined with intravenous GLP-2 in the fasted state [74]. Exendin (9-39) blocks the inhibition of murine small intestinal motility induced by intraduodenal infusion of peptone [75]. Suppression of fasting small intestinal motility by exogenous GLP-1 is also evident in healthy humans and those with irritable bowel syndrome, manifested by a reduction in the frequency of MMCs in a dose-dependent manner [76].Indeed, the GLP-1 analogue, ROSE-010, has been reported to be more effective than placebo at relieving abdominutesal pain in irritable bowel syndrome patients [101]. None of the human studies have hitherto evaluated the effects of GLP-1 or its analogues on postprandial small intestinal motility, but this could represent an additional mode of glucose-lowering by these agents, given that pharmacological inhibition of small intestinal flow events has been shown to reduce the rate of small intestinal glucose absorption in healthy humans [102].

## 6. Effects on Colonic Motility

Only a handful of animal studies have specifically evaluated the effects of GLP-1 (exogenous or endogenous) on colonic motility. Adminutesistration of intra-cerebroventricular GLP-1 was reported to increase rat fecal pellet output and this was reversed by the GLP-1 receptor antagonist, exendin (9-39) [77]. Evidence for a role for GLP-1 in the regulation of colonic transit in humans has been limited to the reports of GLP-1 secreting tumors and their association with severe constipation and markedly delayed colonic transit [78, 79].

## 7. Mechanism of Action of GLP-1 and Incretin-Based Therapies on Gut Motility

The mechanisms by which GLP-1, or incretin-based therapies, exert their motor actions on the gut have not yet been fully elucidated but appear to be complex. A number of studies have indicated involvement of the vagal nerves in mediating some of these effects of GLP-1 [103–105]. Gastric relaxation [68, 105] and postprandial gastric accommodation [68], in response to exogenous GLP-1, are mediated by vagal cholinergic pathways; antro-pyloro-duodenal motility apparently is not [103]. Inhibition of fasting small bowel motility in rats by exogenous GLP-1 is mediated via endogenous nitric oxide (NO), while suppression of fed motility is independent of NO [106]. Studies of the rodent duodenum and colon suggest that GLP-1 can decrease excitatory cholinergic neurotransmission in the enteric nervous system via presynaptic GLP-1 receptors, which modulate NO release [107]. 

Some gastrointestinal motor effects of GLP-1 appear to be centrally mediated—GLP-1 can readily diffuse through the blood-brain barrier [77, 108] to gain access to GLP-1 receptors in the circumventricular organs, the subfornical organ, and area postrema [109]; the latter in particular controls vomiting. Albiglutide (or Albugon) is a newer GLP-1 receptor agonist that does not readily diffuse into the area postrema and has a low prevalence of gastrointestinal adverse effects, possibly for this reason [110, 111].

## 8. Conclusion

Exploiting the properties of GLP-1 to the fullest for therapeutic purposes will require an in-depth understanding, not only of its incretin effects but also of its impact on gut motility. Although the last decade and a half has seen some important steps in that direction, particularly in understanding the impacts of GLP-1 and incretin-based therapies on gastric emptying, it is clearly a work in progress (Table 1). Further research is needed to gain a better understanding of the actions of GLP-1 and incretin-based therapies on small bowel motility, the extent of the role of endogenous GLP-1 on gut function, and how strongly the motor effects of GLP-1-based therapies are maintained with long-term use. The implications of effects on gastric emptying and small intestinal motility for glycaemic control in diabetes are clinically significant, as the former are often disordered in long-standing diabetes.

## Figures and Tables

**Table 1:** Summary of motor effects of GLP-1 and incretin-based therapies on the gastrointestinal tract.

	Gastric motility (delayed gastric emptying)	Small intestinal motility (delayed small intestinal transit)	Large intestinal motility (delayed colonic transit)
*Endogenous GLP-1* *(physiological dose)*	One positive study [73]	No studies available	No studies available
*Exogenous GLP-1* *(pharmacological dose)*	Strong evidence in human studies; healthy [16], obese [69], type 2 diabetic [12], critically ill [70]	Positive evidence in animal studies [74, 75]. Positive effect on fasting motility in humans [76]	Positive evidence in animal studies [77]. Only indirect evidence in humans [78, 79].
*GLP-1 receptor agonists* *(e.g., exenatide, liraglutide)*	Strong evidence with exenatide (healthy) [80], (type 2 diabetes) [80–82]. Some evidence with liraglutide [83, 84]	No studies available	No studies available
*DPP-4 inhibitors* *(e.g., sitagliptin, vildagliptin)*	Positive evidence with animal studies only [85]	No studies available	No studies available
